# Assessment of Peri-Implant Soft Tissues Conditions around Short and Ultra-Short Implant-Supported Single Crowns: A 3-Year Retrospective Study on Periodontally Healthy Patients and Patients with a History of Periodontal Disease

**DOI:** 10.3390/ijerph17249354

**Published:** 2020-12-14

**Authors:** Giorgio Lombardo, Annarita Signoriello, Mauro Marincola, Pier Francesco Nocini

**Affiliations:** 1 Department of Surgery, Dentistry, Paediatrics and Gynaecology (DIPSCOMI), School of Dentistry, University of Verona, Piazzale L.A. Scuro 10, 37134 Verona, Italy; giorgio.lombardo@univr.it (G.L.); pierfrancesco.nocini@univr.it (P.F.N.); 2 Dental Implant Unit, Research Department, Faculty of Dentistry, University of Cartagena, Cartagena 130001, Colombia; mauromarincola@unicartagena.edu.co

**Keywords:** bone loss, mucositis, peri-implantitis, periodontal disease, short, single crown, success, survival, ultra-short

## Abstract

The aim of this retrospective study was to evaluate implant survival, marginal bone loss and peri-implant complications in 326 short and ultra-short implants. Implants were placed in the maxillary and mandibular posterior regions of 140 patients with (PP) and without (NPP) a history of periodontal disease. Clinical and radiographic examinations were performed at 3-year recall appointments. The 8.0, 6.0 and 5.0 mm-length implants placed in PP and NPP were respectively 43.75% and 38.46%, 35.10% and 34.19%, 21.15% and 27.35%; 325 implants (one early failure) were rehabilitated with single crowns in 139 patients. Overall implant survival after 3 years of follow-up was 97.55%, 98.08% and 96.61% for PP and NPP (*p* = 0.46). Crestal bone level variations were not statistically different among PP and NPP; 15.41% of implants presented signs of mucositis, 14.71% and 16.67% in PP and NPP (*p* = 0.64). Setting the threshold for bone loss at 2 mm after 36 months, peri-implantitis prevalence was 2.2%, 1.96% and 2.63% in PP and NPP (*p* = 0.7). Overall implant success was 82.39%, 83.33% and 80.7% for PP and NPP (*p* = 0.55). Short-term outcomes suggest that short and ultra-short locking-taper implants can successfully be restored with single crowns in the posterior jaws both in PP and NPP.

## 1. Introduction

The placement of standard-length implants in conjunction with vertical bone augmentation and major reconstructive procedures usually implies longer treatment times and increased risk of post-operative complications [1]. As implant dimensions have considerably decreased in length and diameter during the last decades [2,3], these drawbacks can be easily avoided by choosing minimally invasive alternative treatments [4], which provide various advantages both for clinicians and patients. On that note, the use of short (length ≥ 6 mm and ≤8 mm) and ultra-short (length ≤ 5 mm) [5] implants in the rehabilitation of extreme maxillary and mandibular atrophies is reported to be as effective as the use of longer implants [6,7,8] in terms of implant survival and bone level stability at medium-term follow-up. On the other hand, recent RCTs (randomized controlled trials) [9] assessed lower cumulative survival rates for short and ultra-short implants, also considering long-term follow-up (at least 5 years) [10]. Furthermore, it is reported [11] that short narrow-diameter implants supporting single-crown can be associated with greater marginal bone loss compared to standard implants.

While outcomes such as implants survival and marginal bone loss were widely evaluated in many studies [12,13,14,15], the influence of biological complications on implants failure was less investigated [16,17]. As implant success directly regards the onset of mucositis and peri-implantitis [18,19,20,21], their prevention and management [22] are essential in long-term maintenance of healthy hard and soft peri-implant tissues. Peri-implant mucositis [23] is characterized by bleeding on gentle probing; erythema, swelling, and/or suppuration may also be present. An increase in probing depth is often observed in the presence of peri-implant mucositis, due to swelling or decrease in probing resistance. It is not possible to define a range of probing depths compatible with peri-implant health, as it can also exist around implants with reduced bone support [23]. There is strong evidence from animal and human experimental studies [23] that plaque is the etiological factor for peri-implant mucositis. Thus, peri-mucositis associated with poor plaque control [24] can be reversed with efficient measures aimed at eliminating the deposits and preventing the development of a subsequent peri-implantitis.

Peri-implantitis [23] is a plaque-associated pathological condition occurring in tissues around dental implants, characterized by inflammation in the peri-implant mucosa and subsequent progressive loss of supporting bone. Peri-implantitis sites exhibit clinical signs of inflammation, bleeding on probing, and/or suppuration, increased probing depths and/or recession of the mucosal margin, in addition to radiographic bone loss (greater than 2 mm [25,26]).

In addition to implant-related and prosthesis-related variables considered for the assessment of implants survival and success, there is an emerging matter about the importance of patient-related factors, such as systemic diseases, smoking [26,27,28,29] and history of periodontal disease. The latter may be considered a preponderant risk factor for the occurrence of peri-implantitis [30,31]. However, the evidence [26,32,33] concerning clinical and radiographic outcomes of short and ultra-short implants placed in patients with treated periodontitis is still scarce, in addition to a lack of homogeneous follow-up terms in the current studies.

The aim of this 3-year retrospective study was to evaluate implant survival, marginal bone loss and implant success in 326 short and ultra-short implants restored with single crowns. The implants were placed in the maxillary and mandibular, edentulous posterior regions of patients with history of periodontal disease (PP), and without history of periodontal disease (NPP).

## 2. Materials and Methods

### 2.1. Study Design and Inclusion Criteria 

In total, 326 implants (191 in the posterior mandible and 135 in the posterior maxilla) placed in 140 patients were included in the study. Patients included in the study had been referred between February 2007 and June 2015 for edentulism (tooth loss caused by trauma, caries or periodontal disease) in the posterior areas of maxilla and mandible at the Dental and Maxillo-Facial Surgery Clinic at the University of Verona (Italy). A 3-year follow-up retrospective study [34,35] was conducted between June and October 2018. The study was approved by the University of Verona Institutional Review Board (Prot. 34934, TISSUESMAXMAND, 30/05/18). The study was conducted (see Appendix A) according to guidelines previously described [34,35].

Patients enrolled for the study matched the following inclusion criteria [34,35]: aged between 18 and 90 years; single-tooth replacement of at least one 8.0, 6.0 or 5.0 mm locking-taper implant supporting a single crown; had no previous consent for bone augmentation procedures; had a history of treated chronic periodontal diseases or never being affected by any forms of periodontal disease; compliance to the regular maintenance program (professional oral hygiene sessions every four months). 

Exclusion criteria comprehended several conditions (see Appendix A), as previously described [34,35].

### 2.2. Surgical Protocol

The implant system used in this study presented specific characteristics [34,35]. Moreover, pre-surgical evaluation was conducted as previously described [34,35] (see Appendix A).

All surgical treatments were carried out by a single clinician. A full-thickness flap was performed, and a high-speed 2.0 mm-diameter pilot drill (with a cutting edge at the apical portion and drilling at 1100 rpm) with external saline irrigation was used to perforate the cortical plate. Final pilot drilling length was determined by measuring residual bone height and adding at least 1.0 mm to the selected implant length to allow for a sub-crestal implant placement. Latch reamers presenting a 0.5 mm progressive increase in diameter were used at 50 rpm, without external irrigation, to widen the osteotomy until the final implant diameter was reached. The selected implant was manually inserted into the osteotomy, a healing plug was placed in the implant well, and autogenous bone collected during the slow speed preparation of the osteotomy was used to fill the gap between the implant and the bony walls. The incisions were closed by single polyglycolic acid sutures (Vycril, ACE Surgical Supply Co., Brockton, MA, USA). A post-operative periapical radiograph was taken, and the patient received post-operative instructions, antibiotic and analgesic prescriptions [34,35].

### 2.3. Prosthetic Protocol and Follow-Up Evaluation

Prosthetic loading and follow-up assessment (see Appendix A) were conducted as previously described [34,35]. The post-surgery evaluation and the follow-up evaluation were respectively performed by other two operators, both different from the clinician who performed the surgical phase.

**Figure 1:**
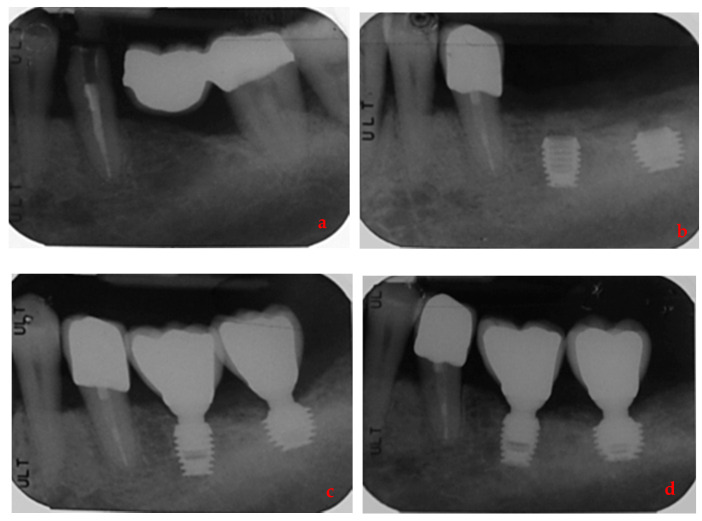
Single implants placed in 3.6 and 3.7 sites (4.5 × 6 mm and 6 × 5 mm) of a male patient with history of periodontal disease: (**a**) pre-operative radiograph before implants placement; (**b**) radiograph obtained at implants placement; (**c**) radiograph obtained at time of loading; (**d**) radiograph obtained at 3-year follow-up.

**Figure 2:**
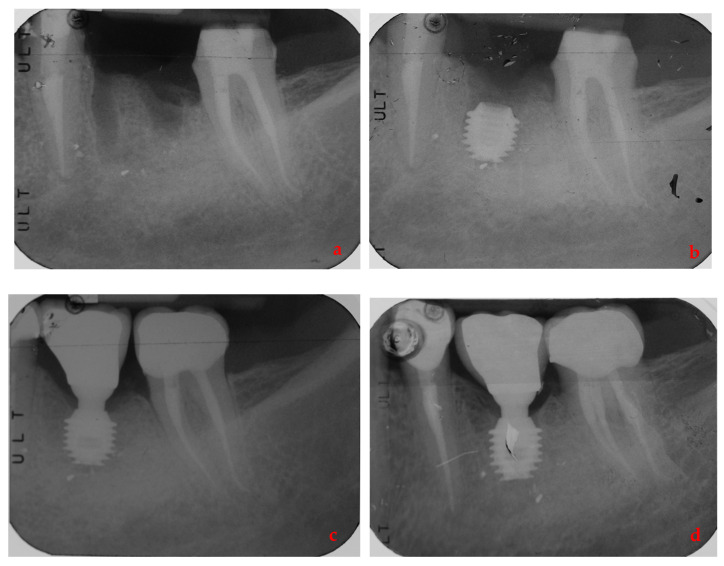
Single implant placed in 3.6 site (5 × 6 mm) of a female patient without history of periodontal disease: (**a**) pre-operative radiograph before implant placement; (**b**) radiograph obtained at implant placement; (**c**) radiograph obtained at time of loading; (**d**) Radiograph obtained at 3-year follow-up.

**Figure 3:**
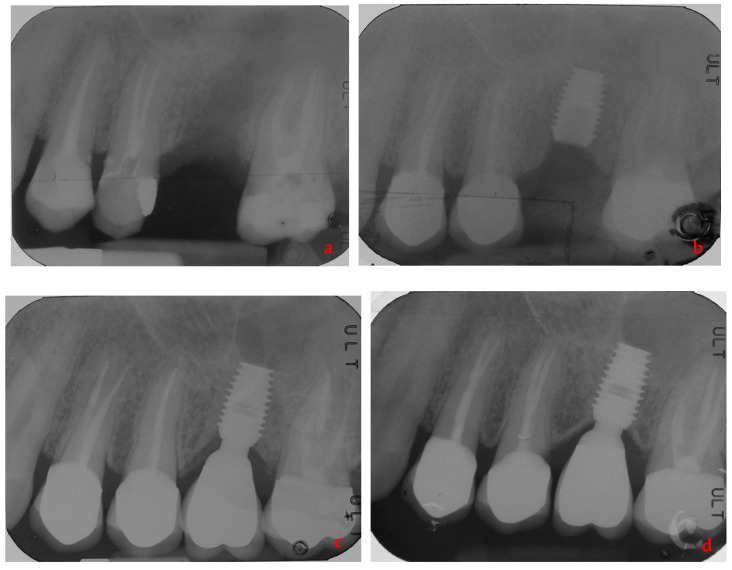
Single implant placed in 2.6 site (4.5 × 8 mm) of a male patient without history of periodontal disease: (**a**) pre-operative radiograph before implant placement; (**b**) radiograph obtained at implant placement; (**c**) radiograph obtained at time of loading; (**d**) radiograph obtained at 3-year follow-up.

**Figure 4:**
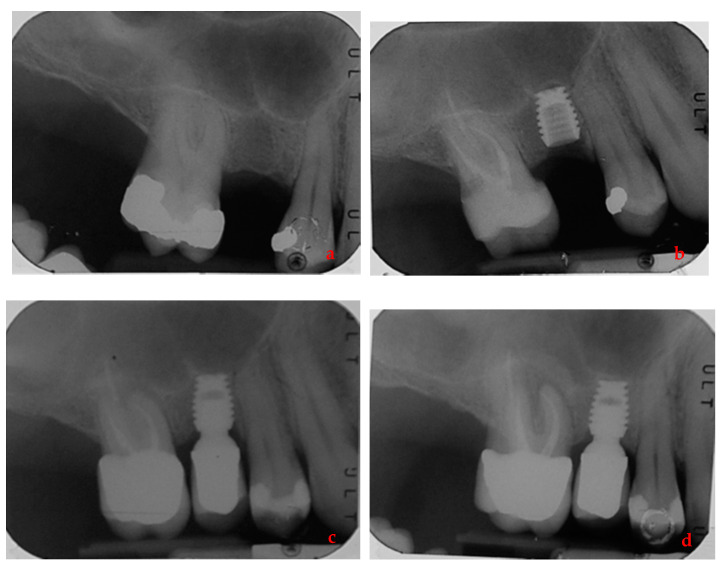
Single implant placed in 1.5 site (4.5 × 6 mm) of a male patient with history of periodontal disease: (**a**) pre-operative radiograph before implant placement; (**b**) radiograph obtained at implant placement; (**c**) radiograph obtained at time of loading; (**d**) radiograph obtained at 3-year follow-up.

By way of illustration, Figure 1, Figure 2, Figure 3 and Figure 4 report some radiographic cases.

### 2.4. Study Variables and Outcomes 

Implant lengths considered in the study were 8.0, 6.0 and 5.0 mm; implant diameters were 3, 3.5, 4.0, 4.5, 5.0, 6.0, and 6.5 mm. Covariates included were: sex, age, smoking history, history of periodontal disease, ASA (American Society of Anesthesiologists) physical status classification, number of oral hygiene sessions per year, use of interproximal oral hygiene devices, arch, tooth site, prosthetic material, crown-to-implant ratio (CIR) [34,35]. Patients with a history of periodontitis (PP) were characterized by previously assessed chronic forms of periodontal disease, corresponding to stage I, II or III, and grade A or B, according to the latest updates on classification of periodontal and peri-implant diseases [24]. PP were subjects following a regular maintenance program on a reduced periodontium, to ensure gingival health at the time of implant placement. On the other hand, periodontally healthy patients (NPP) were subjects never affected by any forms of periodontal diseases.

Study outcomes were implant survival, marginal bone loss and implant success after 3 years of follow-up. Implant survival and marginal bone loss (see Appendix A) were assessed as previously described [34,35].

Peri-implant soft tissues were assessed using a periodontal probe (Florida Probe; Florida Probes Company, Gainesville, FL, USA), applying a force of mild intensity (0.25 N). For each implant site, four parameters were assessed. The Modified Bleeding Index (mBI) and the Modified Plaque Index (mPLI), as reported in the literature by Mombelli [36], were used to record the appropriate values for the mesial, central, and distal on the buccal and lingual/palatal sides of each implant. Similarly, the peri-implant probing depths (PPD) were performed on the same six sites. The amount of keratinized tissue (KT) was assessed by measuring the distance between the zenith of the buccal gingival margin and the mucogingival line [37]. 

Biological complications after loading were also assessed at the 3-year recall appointment. According to the latest updates [23], we defined mucositis as at least one soft-tissue peri-implant surface with positive BOP (bleeding on probing) or pus on probing, PPD ≥ 4 mm and no radiographically detectable bone loss, as it should be noted that visual signs of inflammation can vary and that peri-implant mucositis can exist around implants with variable levels of bone support [24]. We diagnosed peri-implantitis when an implant had simultaneously one surface with positive BOP or pus on probing, increasing PPD compared to previous examinations, and the presence of bone loss beyond crestal bone level changes resulting from initial bone remodeling. In the absence of the previous examination data, diagnosis of peri-implantitis was otherwise based on the combination of presence of positive BOP or pus on probing, PPD ≥ 5 mm [38] and a radiographically detectable bone loss greater than 2 mm [23,24] when compared with the loading measurements.

Implant success was defined according to the following criteria [39,40]: absence of persistent pain, dysesthesia or paraesthesia in the implant area; absence of peri-implant infection with or without suppuration; absence of perceptible mobility of the implant; absence of persistent peri-implant bone resorption >1.5 mm during the first year of loading and >0.2 mm/year during the following years. Once excluded, the failed implants, implant success thus considered implants not presenting signs of mucositis or peri-implantitis.

### 2.5. Statistical Analysis

For data collection, a database including all patients evaluated in the study was created with Microsoft Excel. All data analysis was carried out using Stata v.13.0 for Macintosh (StataCorp, College Station, TX, USA) [41]. Analysis were performed as previously described [34,35] (see Appendix A). 

## 3. Results

### 3.1. Demographics

A total of 140 patients (64 men and 76 women) received at least one 8.0, 6.0 or 5.0 mm-length single-crown dental implant. 78.57% of the patients were non-smokers, 50.71% ASA status I, 55% with history of periodontal disease. All patients were compliant with the maintenance program, following a mean of 2.89 ± 1.19 oral professional hygiene sessions in a year and 74.28% of them used interproximal oral hygiene devices daily. Mean age at placement was 54.14 ± 10.73 (range 28–80) years.

Of implants placed, 136 (41.72%) were 8 mm-length, 114 (34.97%) were 6 mm-length and 76 (23.31%) were 5 mm-length; 191 (58.59%) and 135 (41.41%) implants were respectively positioned in the posterior mandible and maxilla; 208 (63.8%) and 118 (36.2%) implants were respectively positioned in PP and NPP. One implant in the posterior upper maxilla failed before loading, thus 325 implants in 139 patients (63 men and 76 women) were finally rehabilitated with single crowns. The mean CIR was 1.92 ± 0.52 (range 0.91–4.1) and 51.69% of the implants presented a CIR ≥2. CIR in PP and NPP was, respectively, 1.94 ± 0.55 (range 0.91–4.1) and 1.88 ± 0.45 (range 1.09–3.1), with no statistically significant differences among groups (*p* = 0.58). The loaded implants distribution, analyzed according to PP and NPP, is presented in Table 1. 

### 3.2. Implant Survival and Marginal Bone Loss

One early failure was assessed, and seven implants were lost and removed after functional loading in seven different patients. The overall implant survival at the 36-month follow-up was 97.55% (318/326). Failures features are recorded in Table 2.

No association was found between survival and failure groups, and any of the covariates considered (Table 3).

The implant survival according to length-groups was 97.79% for 8 mm-length implants, 97.37% for 6 mm-length implants, 97.37% for 5 mm-length implants, respectively. According to arch-groups, 97.38% of the implants in the posterior mandible and 97.78% in the posterior maxilla survived. In regard to history of periodontal disease, 208 implants placed in patients with a history of chronic periodontitis presented a survival of 98.08%, while 118 implants placed in patients who had no history of periodontal disease, but lost their teeth for other reasons, presented a survival of 96.61%. No statistically significant differences after 3 years of follow-up were found between length-groups (*p* = 0.97), arch-groups (*p* = 0.56) or PP/NPP-groups (*p* = 0.46).

ΔCBL (average bone loss) and ΔF-BIC (average apical shift of the “first bone-to-implant contact point” position), compared by one-way non-parametric analysis of variance (ANOVA) with each covariate as between-patients factor, were not statistically different among length-groups, arch-groups or PP/NPP-groups after 3 years of follow-up. Crestal bone level variations are reported in Table 4.

### 3.3. Soft Tissues’ Conditions and Implant Success

No statistically significant differences in soft tissues’ conditions (PPD, mBI, mPLI and KT), at 3-year recall appointment, were found between length-groups, arch-groups or PP/NPP-groups (Table 5), except for KT values between length-groups (*p* < 0.001).

Among 318 survived implants at the 3-year follow-up, 49 (15.41%) exhibited peri-mucositis and 7 (2.2%) presented peri-implantitis, for a total of 56 implants (17.61%) presenting biological complications. A statistically greater prevalence of peri-mucositis (*p* < 0.01) was found in the posterior mandible compared to the posterior maxilla (Table 6 and Table 7).

The overall implant success at 36-month follow-up (Table 8) was 82.39% (262/318): 87.22% for 8.0 mm-length implants, 81.98% for 6.0 mm-length implants, 74.32% for 5.0 mm-length implants respectively (*p* = 0.06); 77.42% for posterior mandible and 89.39% for posterior maxilla (*p* < 0.01); 83.33% for PP and 80.7% for NPP (*p* = 0.55).

No associations were found between peri-mucositis or peri-implantitis and any of the covariates considered, except for the number of oral professional hygiene/year (*p* = 0.01) related to peri-mucositis insurgence.

## 4. Discussion

Peri-implantitis is defined as inflammation of the peri-implant mucosa, plaque association and non-reversible, radiographically detectable bone loss that exceeds normal physiological remodelling [42]. This condition, in the absence of treatment, seems to progress in a non-linear and accelerating pattern [19,43,44]. A strong similarity between the bacterial composition of sites with periodontitis and sites with peri-implantitis has been observed [45,46,47]. This could be considered a crucial point in endorsing the implant placement in patients without a history of periodontal disease, in order to avoid the possibility of serious peri-implant complications. Furthermore, residual pockets at the end of active periodontal therapy represent a significant risk for the development of peri-implant bone loss in patients susceptible to periodontitis [48], even if the patient is compliant to an established maintenance protocol.

Current reported prevalence of peri-implant diseases is not unequivocally determined in literature [23,26,49,50], because of multiple discrepancies regarding different definition, implant-related characteristics, prosthetic protocols and bone loss threshold indicative of destructive process. A systematic review based on an average follow-up of 3 years [20] reported an implant-based prevalence of peri-implant mucositis and peri-implantitis of 29.48% and 9.25% respectively.

Nevertheless, recent studies showed that implants placed in NPP demonstrate fewer failures, and consequent higher percentages of implant survival, compared to those placed in PP. Karoussis et al. stated [51] that implants in patients with history of periodontitis usually encounter less survival (90.5%) compared to implants in patients with no past history of periodontitis (96.5%) after a long-term follow-up. Hardt et al. [52] considered 346 implants placed in the posterior maxillary areas with a follow-up of 5 years: the survival was 96.7% and 92% for NPP and PP respectively. Roccuzzo et al. [53] found a 10-year survival rate of 96.6%, 92.8% and 90% for 61, 95 and 90 implants placed respectively in periodontally healthy patients, patients with a history of moderate periodontitis and patients with a history of severe periodontitis.

Concerning the increased risk for developing peri-implantitis due to the susceptibility to periodontitis, stated by many authors [26,50,54,55,56,57], Changi et el. [50], in a 3.5-year study on 6129 implants, demonstrated that radiographic evidence of periodontitis is one of the principal risk-factor statistically associated (odds ratio (OR) = 3.6) with peri-implantitis. Renvert et al. [57] found a OR even equal to 4.5 assessing the likelihood of association between peri-implantitis and history of periodontitis. Moreover, insurgence of peri-implantitis seem to be higher in PP: Karoussis et al. considered [51] 112 ITI dental implants, comparing 21 implants placed in PP and 91 implants in NPP, both following regular supportive therapy for 10 years, and found that incidence of peri-implantitis in NPP (5.8%) was lower compared to PP (28.6%). In a 3- to 5-year cross-sectional study, Arunyanak et al. found [58] that prevalence of peri-implantitis was significantly higher in PP (25% in 72 patients) compared to NPP (10.9% in 128 patients).

On the other hand, investigations involving short implants (length ≥6 mm and ≤8 mm) and considering a history of periodontal disease as a variable with potential correlation with failure and biological complications, are still scarce in literature. Hasanoglu et al., [33] in a multicenter long-term retrospective study on 460 short implants (4 to 9 mm in length) placed both in posterior and anterior regions of maxilla and mandible of 299 patients, found an overall implant survival of 95.86% and a prevalence of peri-implantitis of 10% after a follow-up of up to 9 years, with 73.91% of failures caused by peri-implantitis; in this study, 70.85% of implants were placed in patients without a history of periodontal disease. Zhang et al. [59], in a study on 214 implants, whose length was less than 8 mm in 25 implants, assessed implant-related variables (e.g., length, diameter and position) and periodontal-related variables (e.g., soft tissue indexes and marginal bone-level alterations), identifying residual pockets and posterior region as predictors for peri-implantitis. Akram et al. [32], in a 3-year follow-up study, compared the clinical and radiographic conditions between teeth of healthy patients (11) and short implants placed in patients treated for aggressive periodontitis (48); soft tissues parameters of PI, BOP, PD and CAL were recorded, finding a significantly greater attachment loss in implants compared to teeth. Correia et al. [60], in a retrospective study on 689 implants in 202 patients, found an overall implant survival of 95.8% for NPP (214 implants) and 93.1% for PP (475 implants), after 3 years of follow up, with no statistically significant differences between groups; moreover, short implants showed a survival of 97.3% and 93% for NPP (74 implants) and PP (157 implants) respectively, with no statistically significant differences between groups.

Similarly, in this 3-year retrospective study, a history of periodontitis seemed not to be correlated to implant failure, as no statistically significant differences in implant survival were found between PP and NPP.

Excessive bone loss after loading can influence both implant survival and success: our results showed that bone level stability was preserved after 3 years, without significant differences between implant placed in PP and NPP. It is also worth noting that the implant system examined in the study presents a screw-less locking-taper implant-abutment connection, which increases mechanical stability with no micromovements or micro-gaps at the implant-abutment interface and provides minimal bone resorption [61]. Moreover, the convergent crest module in short and ultra-short implants seems to have an important influence on marginal bone loss. Referring to biomechanical models which compare different crest modules, the quantity of bone present around the neck of the implant is fundamental for the distribution of the occlusal forces [62,63]. The transmission of vertical, horizontal and rotational forces on F-BIC is thus more favorable and homogeneous in implants with convergent crest module compared to implants with divergent crest module with the same diameter. Furthermore, the sloping shoulder guarantees a platform switching at implant level with bone growth over the neck, assuring successful long-term functioning together with the specific plateau root-form design [62].

The literature supports a general agreement that implants can be successfully placed in periodontal patients if proper supportive protocols of maintenance are applied before and after loading [64,65], in order to prevent peri-implant mucositis and peri-implantitis. Some authors [66] claimed that current definitions of peri-implant health and diseases are still greatly debated and controversial, as healthy implant mucosa may bleed upon probing, thus leading to high number of false-positives. Nevertheless, an increase of probing pocket depth values over time is not necessarily associated with loss of supporting bone around dental implants. It is also suggested that bleeding on probing should be used as a diagnostic tool and as an indicator for treatment in association with probing pocket depth of at least ≥4 mm, the presence of abundant plaque deposits, and radiographic detection of bone loss [66]. Furthermore, the evidence is equivocal regarding the effects of keratinized mucosa (which was statistically different between length-groups in our study) on the long-term health of the peri-implant tissue, such as patient comfort and ease of plaque removal [23].

In the present study, where patients adhered to a strictly observed protocol of TPS, low inflammatory indexes were generally assessed, with a positively significant correlation with the number of oral hygiene interventions administered per year. Finally, only 15.41% of the implants presented signs of mucositis, with no statistically significant differences between PP and NPP. Similar results were found in a study by Zorzano et al. [67], where 786 implants were placed in 239 periodontally compromised patients, who regularly received supportive periodontal therapy; after a mean follow-up of 63 months, 12.8% of the implants were affected by peri-mucositis and 9.8% by peri-implantitis.

However, the present study, being retrospective, presents some critical issues. The medium sample size, the relatively short evaluation (3 years of follow-up) and a non-homogeneous distribution among implant length-groups, arch-groups and PP/NPP-groups are the product of its retrospective nature. The single-center setting, involving a university dental clinic, could also have introduced an important bias, suggesting that our results cannot be generalized.

Another issue that could represent a critical limit for the study is that most of the implants were placed in patients characterized by a history of periodontal disease: nonetheless, after 3 years of loading, the main strengths of our study rests on a positive assessment of the proportion of surviving implants and bone level stability when placing short and ultra-short single-crown locking-taper implants both in PP and NPP. Furthermore, all patients enrolled in the study showed a positive compliance to the maintenance program.

Prospective long-term (5-year follow-up or longer) studies are necessary for a better evaluation of larger homogenous samples, and a more balanced distribution between patients with and without a history of periodontal disease is desirable.

## 5. Conclusions

Short-term outcomes suggest that short and ultra-short locking-taper implants can be successfully placed and restored with single crowns in the atrophic posterior jaws both in PP and NPP. By contrast with several studies in the literature, our outcomes showed that a history of periodontal disease does not seem to negatively influence peri-implant conditions. Suitable maintenance procedures before implant placement and during the follow-up time, together with adequate compliance of the patients in daily homecare, mainly contributed to our stable results, both for PP and NPP. Further investigations with longer follow-up are, of course, necessary to validate these conclusions.

## Figures and Tables

**Table 1:** Overall loaded implants: PP/NPP-groups distribution according to study variables. PP = patients with history of periodontal disease; NPP = patients without history of periodontal disease; ASA = American Society of Anesthesiologists physical status classification. Age at follow-up and oral professional hygiene/year are presented as mean ± standard deviation; for all other variables, values are presented as n (%); NS = not statistically significant; d.f. = degrees of freedom.

Variable	NPP	PP	Test Statistic	d.f.	*p* Value
**Sex**					
male	53 (45.30)	89 (42.79)	χ^2^ = 0.19	1	NS (*p* = 0.66)
female	64 (54.70)	119 (57.21)			
**Age at follow-up**	55.41 ± 10.56	60.56 ± 9.95	F = 19.18	1/325	<0.001
**Smoking history**					
no	87 (74.36)	172 (82.69)	χ^2^ = 3.21	1	NS (*p* = 0.07)
yes	30 (25.64)	36 (17.31)			
**ASA status**					
I	65 (55.56)	80 (38.46)	χ^2^ = 9.03	1	0.01
II	52 (44.44)	128 (61.54)			
**Oral professional hygiene/year**	2.88 ± 1.34	2.89 ± 1.11	F = 14.31	1/325	NS (*p* = 0.92)
**Use of interproximal oral hygiene devices**					
no	30 (25.64)	51 (24.52)	χ^2^ = 0.05	1	NS (*p* = 0.82)
yes	87 (74.36)	157 (75.48)			
**Implant length**					
5 mm	32 (27.35)	44 (21.15)	χ^2^ = 1.74	2	NS (*p* = 0.41)
6 mm	40 (34.19)	73 (35.10)			
8 mm	45 (38.46)	91 (43.75)			
**Implant tooth site**					
premolar	46 (39.32)	98 (47.12)	χ^2^ = 1.84	1	NS (*p* = 0.17)
molar	71 (60.68)	110 (52.88)			
**Arch**					
posterior mandible	68 (58.12)	123 (59.13)	χ^2^ = 0.03	1	NS (*p* = 0.85)
posterior maxilla	49 (41.88)	85 (40.87)			
**Implant diameter**					
3 mm	1 (0.85)	0 (0.00)	χ^2^ = 5.75	6	NS (*p* = 0.44)
3.5 mm	6 (5.13)	7 (3.37)			
4 mm	28 (23.93)	61 (29.33)			
4.5 mm	39 (33.33)	76 (36.54)			
5 mm	36 (30.77)	55 (26.44)			
6 mm	6 (5.13)	9 (4.33)			
6.5 mm	1 (0.85)	0 (0.00)			
**Prosthetic material**					
resin	18 (15.38)	35 (16.83)	χ^2^ = 0.11	1	NS (*p* = 0.73)
porcelain	99 (84.62)	173 (83.17)			
**Crown-to-implant ratio (CIR)**					
<2	68 (58.12)	89 (42.79)	χ^2^ = 7.75	2	0.02
2 < CIR < 2.99	48 (41.03)	113 (54.33)			
>2.99	1 (0.85)	6 (2.88)			

**Table 2:** Failures features.

**Site**	45	46	24	47	16	34	44	17
**Diameter**	6	4.5	4	5	5	4.5	5	5
**Length**	5	8	5	6	6	8	8	6
**Sex**	male	male	female	female	male	male	male	male
**Smoking history**	no	no	no	no	yes	yes	no	no
**ASA status**	I	I	I	II	II	II	II	I
**Oral professional hygiene/year**	3	4	4	4	3	2	4	4
**History of periodontal disease**	yes	no	no	no	yes	yes	yes	no
**Crown-to-implant ratio**	2.76	1.38	2.25	2.17	2.68	1.69	1.62	/
**Failure**	late	late	late	late	late	late	late	early

**Table 3:** Analysis of implant survival according to study covariates included. For all variables, values are presented as n (%); NS = not statistically significant; d.f. = degrees of freedom.

Variable	Survival	Failure	Test Statistic	d.f.	*p* Value
	n (%)	n (%)			
**Sex**					
male	137 (95.80)	6 (4.20)	χ^2^ = 3.22	1	NS (*p* = 0.14)
female	181 (98.91)	2 (1.09)			
**Smoking history**					
no	254 (97.69)	6 (2.31)	χ^2^ = 0.11	1	NS (*p* = 0.66)
yes	64 (96.97)	2 (3.03)			
**ASA status**					
I	142 (97.26)	4 (2.74)	χ^2^ = 4.41	1	NS (*p* = 0.11)
II	176 (97.78)	4 (2.22)			
**History of periodontal disease**					
no	114 (96.61)	4 (3.39)	χ^2^ = 0.67	1	NS (*p* = 0.46)
yes	204 (98.08)	4 (1.92)			
**Implant tooth site**					
premolar	140 (97.22)	4 (2.78)	χ^2^ = 0.11	1	NS (*p* = 0.73)
molar	178 (97.80)	4 (2.20)			
**Arch**					
posterior mandible	186 (97.38)	5 (2.62)	χ^2^ = 0.05	1	NS (*p* = 0.56)
posterior maxilla	132 (97.78)	3 (2.22)			
**Implant diameter**					
3 mm	1 (100.00)	0 (0.00)			
3.5 mm	13 (100.00)	0 (0.00)			
4 mm	88 (98.88)	1 (1.12)	χ^2^ = 3.77	6	NS (*p* = 0.41)
4.5 mm	113 (98.26)	2 (1.74)			
5 mm	88 (95.65)	4 (4.35)			
6 mm	14 (93.33)	1 (6.67)			
6.5 mm	1 (100.00)	0 (0.00)			
**Implant length**					
8 mm	133 (97.79)	3 (2.21)	χ^2^ = 0.06	2	NS (*p* = 0.97)
6 mm	111 (97.37)	3 (2.63)			
5 mm	74 (97.37)	2 (2.63)			
**Prosthetic material**					
resin	53 (100)	0 (0.00)	χ^2^ = 1.39	1	NS (*p* = 0.60)
porcelain	265 (97.43)	7 (2.57)			
**Crown-to-implant ratio**					
<2	154 (98.09)	3 (1.91)	χ^2^ = 0.33	2	NS (*p* = 0.76)
2–2.99	157 (97.52)	4 (2.48)			
>2.99	7 (100.00)	0 (0.00)			

**Table 4:** Overall CBL/F-BIC (crestal bone level/first bone-to-implant contact) distributions and analysis of ΔCBL/ΔF-BIC according to PP/NPP-groups, length-groups and arch-groups. CBL/F-BIC and its variations are presented as median [iqr, interquartile range]; NS = not statistically significant; d.f. = degrees of freedom.

Variable	Overall	History of Periodontal Disease		Implant Length			Arch	
		no	yes	5 mm	6 mm	8 mm	Posterior Mandible	Posterior Maxilla
**CBL**								
Loading time	1.97	2.17	1.93	1.82	1.87	2.20	2.10	1.88
[median (iqr)]	(1.64)	(1.53)	(1.63)	(1.40)	(1.83)	(1.71)	(1.62)	(1.63)
Follow-up time	1.57	1.57	1.58	1.42	1.55	1.78	1.59	1.55
[median (iqr)]	(1.8)	(1.67)	(1.86)	(1.75)	(1.97)	(1.86)	(1.78)	(1.90)
**Δ** **CBL**	0.36	0.41	0.34	0.32	0.36	0.36	0.33	0.45
[median (iqr)]	(1.03)	(1.08)	(0.95)	(1.05)	(0.96)	(1.11)	(1.00)	(0.99)
test statistic								
d.f.		Z = 0.38			χ^2^ = 0.23			Z = −0.82
*p* value					2			
		NS (*p* = 0.69)			NS (*p* = 0.89)			NS (*p* = 0.40)
**F-BIC**								
Loading time	0.22	0.01	0.28	0.37	0.18	0.18	0.23	0.21
[median (iqr)]	(0.66)	(0.54)	(0.70)	(0.71)	(0.62)	(0.65)	(0.68)	(0.66)
Follow-up time	0.40	0.44	0.38	0.53	0.39	0.33	0.43	0.38
[median (iqr)]	(0.84)	(0.85)	(0.81)	(0.73)	(0.85)	(0.72)	(0.89)	(0.74)
**Δ** **F-BIC**	0.05	0.14	0.01	0.19	0.09	0.01	0.09	0.01
[median (iqr)]	(0.56)	(0.46)	(0.57)	(0.56)	(0.63)	(0.41)	(0.46)	(0.60)
test statistic								
d.f.		Z = 1.73			χ^2^ = 2.77			Z = 1.67
*p* value					2			
		NS (*p* = 0.08)			NS (*p* = 0.24)			NS (*p* = 0.09)

**Table 5:** Overall soft tissues indices (Modified Bleeding Index (mBI), Modified Plaque Index (mPLI), peri-implant probing depths (PPD) [mm], keratinized tissue (KT) [mm]) according to PP/NPP-groups, length-groups and arch-groups. mBI, mPLI, PPD and KT are presented as mean ± standard deviation; for all other variables, values are presented as n (%); NS = not statistically significant; d.f. = degrees of freedom.

**Variable**	**mBI**	**Test Statistic**	**d.f.**	***p* Value**
	**[mean ± sd]**			
**Overall**	0.9 ± 0.8			
**History of periodontal disease**				
no	0.9 ± 0.83	Z = −0.08		NS (*p* = 0.93)
yes	0.9 ± 0.79			
**Arch**				
posterior mandible	0.91 ± 0.82	Z = 0.16		NS (*p* = 0.87)
posterior maxilla	0.88 ± 0.78			
**Implant length**				
8 mm	0.86 ± 0.75			
6 mm	0.92 ± 0.79	χ^2^ = 0.20	2	NS (*p* = 0.90)
5 mm	0.94 ± 0.92			
**Variable**	**mPLI**	**Test Statistic**	**d.f.**	***p* Value**
	**[mean ± sd]**			
**Overall**	0.52 ± 0.73			
**History of periodontal disease**				
no	0.56 ± 0.79	Z = 0.23		NS (*p* = 0.81)
yes	0.5 ± 0.69			
**Arch**				
posterior mandible	0.5 ± 0.72	Z = −0.77		NS (*p* = 0.43)
posterior maxilla	0.55 ± 0.74			
**Implant length**				
8 mm	0.48 ± 0.67			
6 mm	0.49 ± 0.72	χ^2^ = 1.89	2	NS (*p* = 0.38)
5 mm	0.64 ± 0.82			
**Variable**	**PPD**	**Test Statistic**	**d.f.**	***p* Value**
	**[mean ± sd]**			
**Overall**	3.29 ± 1.28			
**History of periodontal disease**				
no	3.35 ± 1.4	Z = 0.18		NS (*p* = 0.85)
yes	3.26 ± 1.2			
**Arch**				
posterior mandible	3.29 ± 1.41	Z = −0.68		NS (*p* = 0.49)
posterior maxilla	3.29 ± 1.07			
**Implant length**				
8 mm	3.33 ± 1.35			
6 mm	3.23 ± 1.08	χ^2^ = 0.12	2	NS (*p* = 0.93)
5 mm	3.32 ± 1.42			
**Variable**	**KT**	**Test Statistic**	**d.f.**	***p* Value**
	**[mean ± sd]**			
**Overall**	2.47 ± 1.69			
**History of periodontal disease**				
no	2.49 ± 1.79	Z = 0.19		NS (*p* = 0.84)
yes	2.45 ± 1.63			
**Arch**				
posterior mandible	2.41 ± 1.69	Z = −0.70		NS (*p* = 0.48)
posterior maxilla	2.54 ± 1.68			
**Implant length**				
8 mm	3.2 ± 1.63			
6 mm	1.8 ± 1.41	χ^2^ = 44.52	2	<0.001
5 mm	2.14 ± 1.68			

**Table 6:** Prevalence of peri-mucositis according to PP/NPP-groups, length-groups and arch-groups. For all variables, values are presented as n (%); NS = not statistically significant; d.f. = degrees of freedom.

Variable	No Peri-Mucositis	Peri-Mucositis	χ^2^	d.f.	*p* Value
	n (%)	n (%)			
**History of periodontal disease**					
no	95 (83.33)	19 (16.67)	0.21	1	NS (*p* = 0.64)
yes	174 (85.29)	30 (14.71)			
**Arch**					
posterior mandible	148 (79.57)	38 (20.43)	8.66	1	<0.01
posterior maxilla	121 (91.67)	11 (8.33)			
**Implant length**					
8 mm	118 (88.72)	15 (11.28)	3.98	2	NS (*p* = 0.13)
6 mm	93 (83.78)	18 (16.22)			
5 mm	58 (78.38)	16 (21.62)			

**Table 7:** Prevalence of peri-implantitis according to PP/NPP-groups, length-groups and arch-groups. For all variables, values are presented as n (%); NS = not statistically significant; d.f. = degrees of freedom.

Variable	No Peri-Implantitis	Peri-Implantitis	χ^2^	d.f.	*p* Value
	n (%)	n (%)			
**History of periodontal disease**					
no	111 (97.37)	3 (2.63)	0.15	1	NS (*p* = 0.70)
yes	200 (98.04)	4 (1.96)			
**Arch**					
posterior mandible	182 (97.85)	4 (2.15)	0.005	1	NS (*p* = 0.61)
posterior maxilla	129 (97.73)	3 (2.27)			
**Implant length**					
8 mm	131 (98.50)	2 (1.50)	1.56	2	NS (*p* = 0.49)
6 mm	109 (98.20)	2 (1.80)			
5 mm	71 (95.95)	3 (4.05)			

**Table 8:** Implant success according to PP/NPP-groups, length-groups and arch-groups. For all variables, values are presented as n (%); NS = not statistically significant; d.f. = degrees of freedom.

Variable	Success	No Success	χ^2^	d.f.	*p* Value
	n (%)	n (%)			
**History of periodontal disease**					
no	92 (80.70)	22 (19.30)	0.34	1	NS (*p* = 0.55)
yes	170 (83.33)	34 (16.67)			
**Arch**					
posterior mandible	144 (77.42)	42 (22.58)	7.63	1	<0.01
posterior maxilla	118 (89.39)	14 (10.61)			
**Implant length**					
8 mm	116 (87.22)	17 (12.78)	5.46	2	NS (*p* = 0.06)
6 mm	91 (81.98)	20 (18.02)			
5 mm	55 (74.32)	19 (25.68)			

## Appendix A

### Appendix A.1. Study Design and Inclusion Criteria

The nature and aim of the study, together with the anonymity in the scientific use of data, were clearly presented in a written informed consent form, and signed by every patient. All procedures accorded with Helsinki Declaration and good clinical practice guidelines for research on human beings [34,35].

Exclusion criteria for the study were [34,35]: presence of active infection at an implant site; ASA status III (according to the American Society of Anesthesiologists classification), that is severe systemic diseases or substantive functional limitations which contraindicated implant surgery (such as drug or alcohol abuse, uncontrolled diabetes mellitus, immunosuppression or immunodepression, severe autoimmune diseases, treatment or past treatment with intravenous amino-bisphosphonates for metastatic bone diseases, radiotherapy to head or neck within two years prior to treatment, history of malignancy or chemotherapy within the previous year, treatment with oral amino-bisphosphonates for more than three years, morbid obesity, active hepatitis, severe renal disease, severe cardiovascular conditions, recent history of myocardial infarction (MI) or transient ischemic attack (TIA)); ASA status IV, V, and VI; untreated periodontitis; poor oral hygiene and motivation; current pregnancy or lactation; heavy smoking (more than 25 cigarettes per day); severe clenching or bruxism.

### Appendix A.2. Surgical Protocol

The locking-taper (Morse taper or Morse cone) dental implant system (Bicon Dental Implants, Boston, MA, USA, designed in 1985) used in this study presents an implant interface connection to its restoration, which is impervious to bacterial penetration or infiltration [68]. The implant system also includes a convergent crest module, platform switching, plateau root-form design, and an Integra CP surface (hydroxyapatite treated and acid-etched) [34,35].

A complete clinical and radiographic evaluation (dental and periodontal status; panoramic and periapical radiograph, cone beam computed tomography) and basic periodontal treatment was performed before implant placement. A pre-operative medication consisting of 2 g of Augmentin (875 mg amoxicillin plus 125 mg clavulanic acid), or 1 g of Klacid (Clarithromycin 500 mg) if allergic to penicillin, was given one hour before surgery. All surgical procedures were performed under local anaesthesia, using only Articain 4% with adrenaline 1:100,000 (Citocartin) or Articain 4% with adrenaline 1:100,000 (Citocartin) associated with oral sedation (Halcion 0.25 mg) [34,35].

### Appendix A.3. Prosthetic Protocol and Follow-Up Evaluation

After 4 to 6 months the implants were surgically uncovered, and the healing abutments placed, readapting the mucosal flaps around them. After three weeks of soft tissue healing, impressions were taken using a polyether material (3M ESPE Impregum Impression Material). Definitive single-crown porcelain or composite restorations were delivered within two weeks. The choice for restorative materials (porcelain or composite) was based on patients’ preference, guided by personal economic resources in most of the cases. The technique used for the composite restorations was the Integrated Abutment Crown (IAC), in which the abutment and the crown material are extra-orally chemo-mechanically bonded; therefore, there is no need for cement, and the implant and implant-abutment are connected with a screwless locking-taper connection [34,35,69].

Recall appointments were established to manage prosthetic complications as needed. A maintenance program was designed in order to provide patients a professional oral hygiene session every four months. Clinical and radiographic examinations were performed during the follow-up 3 years from loading time [34,35].

### Appendix A.4. Study Variables and Outcomes

In regard to implant survival, failure was considered as the need for implant removal either before loading (due to no osseointegration) or after loading (due to excessive bone loss). Implant survival was thus considered as the implant’s state of being in function at the three-year follow-up evaluation, that is, symptom-free, without mobility, radiolucency, or bone loss so severe as to warrant implant removal [12,34,35,70,71].

A descriptive analysis of crestal bone level (CBL, average bone level around implants at mesial and distal sides, in mm) and first bone-to-implant contact (F-BIC, in mm) [72,73,74] along with their variations ΔCBL (average bone loss) and ΔF-BIC (average apical shift of the “first bone-to-implant contact point” position), was conducted between loading time and the 3-year follow-up time, according to covariates. Peri-implant bone levels were measured through digitally scanned intraoral radiographs, performed with a paralleling technique [75], using Rinn centering devices (Rinn XCP Posterior Aiming Ring-Yellow, Dentsply, Elgin, IL, USA), immediately after implant placement, at healing abutment placement, at prosthetic loading, and after 3 years of loading. The implant–abutment interface (IAI) was taken as a reference for measurements. CBL was measured on mesial and distal sides as the linear distance between the IAI and the highest point of the interproximal bone crest parallel to the lateral sides of the implant body: a positive value was given when the crest was located coronally to the IAI and a negative value when the crest was located apically to the IAI. For every implant, at each examination interval, an average mesial-distal value was calculated. F-BIC was defined as the first most coronal bone-to-implant relationship visible at the first line of contact, on both mesial and distal sides; if F-BIC matches with IAI, the measurement was 0; if it is located apically, the measurement was a positive value [34,35]. As described in the literature [76], implants were divided into two groups on the basis of presenting a CIR less than or greater than two. The crown height was measured on the radiograph immediately after the prosthetic loading, from the most occlusal point to the IAI. Anatomical crown-to-implant ratio (in which the fulcrum is positioned at the interface between the implant shoulder and the crown-abutment complex) was calculated by dividing the digital length of the crown by the digital length of the implant [34,35].

Measurements were assessed with the aid of a software program (Rasband, W.S., ImageJ, U. S. National Institutes of Health, Bethesda, Maryland, USA) which uses a measuring tool in conjunction with a magnification tool. To correct the distortion of the radiographic image, the apparent size of each implant (measured directly on the radiograph) was compared with the actual length to determine, with adequate precision, the amount of any changes of the crestal bone around each implant. The measurements were made to the nearest 0.01 mm. One dentist who was not involved in the treatment of the patients completed all the measurements on periapical radiographs; the observation intervals of radiographs were masked to the examiner. Before the start of the study, this investigator was calibrated for intra-examiner adequate levels of accuracy and reproducibility in recording the radiographic parameters. Three radiographs were enrolled for this purpose: duplicate measurements for CBL, F-BIC and CIR were collected with an interval of 24 h between the first and second recording. The intra-class correlation coefficients, used as a measure of intra-examiner reproducibility, had to be greater than 0.8 [34,35].

### Appendix A.5. Statistical Analysis

The normality assumptions for continuous data were assessed by using the Shapiro–Wilk test; mean and standard deviation were reported for normally distributed data, median and interquartile range (iqr) otherwise. For categorical data, absolute frequencies, percentages and 95% confidence intervals were reported. The association between categorical variables was tested with χ^2^ test; if any of the expected values was less than 5, a Fisher’s exact test was performed. The comparison between the means of continuous variables in two different times was performed by using paired Student’s “t” test or Wilcoxon matched-pairs signed-rank test. The comparison between the means of two different groups was performed using unpaired Student’s “t”, or Wilcoxon rank-sum test. The comparison of the means among more than two groups was undertaken using one-way analysis of variance (ANOVA), or the Kruskal–Wallis equality-of-populations rank test. Significance level was set at 0.05 [34,35].
